# 16S rRNA sequencing of samples from universal stool bank donors

**DOI:** 10.1186/s13104-021-05520-z

**Published:** 2021-03-23

**Authors:** Marina Santiago, Scott W. Olesen

**Affiliations:** OpenBiome, 2067 Massachusetts Ave, Cambridge, MA 02140 USA

**Keywords:** Human microbiome, 16S rRNA sequencing, Stool donor, Fecal microbiota transplant, Donors, Stool sample, Feces

## Abstract

**Objectives:**

Universal stool banks provide stool to physicians for use in treating recurrent *Clostridioides difficile* infection via fecal microbiota transplantation. Stool donors providing the material are rigorously screened for diseases and disorders with a potential microbiome etiology, and they are likely healthier than the controls in most microbiome datasets. 16S rRNA sequencing was performed on samples from a selection of stool donors at a large stool bank, OpenBiome, to characterize their gut microbial community and to compare samples across different timepoints and sequencing runs.

**Data description:**

16S rRNA sequencing was performed on 200 samples derived from 170 unique stool donations from 86 unique donors. Samples were sequenced on 11 different sequencing runs. We are making this data available because rigorously screened, likely very healthy stool donors may be useful for characterizing and understanding microbial community differences across different populations and will help shed light into the how the microbiome community promotes health and disease.

## Objective

Universal stool banks provide rigorously-screened stool to physicians treating patients with recurrent *Clostridioides difficile* infection using fecal microbiota transplantation under US Food and Drug Administration enforcement discretion [1, 2] as well as for research purposes. These stool banks provide centralized donor screening and material preparation, which increases the quality and accessibility of fecal microbiota transplantation as a therapy. Rigorous screening of donors is required to prevent transmission of pathogens or other microbiome-mediated diseases from the donor to the recipient.

**Table 1:** Overview of data files/datasets

Label	Name of data file/dataset	File types (file extension)	Data repository and identifier (DOI or accession number)
Data set 1	Raw sequencing files	.fastq	*European Nucleotide Archive*; https://identifiers.org/ena.embl:PRJEB41316
Data file 1	exclusion_criteria_comparison	.xlsx	*Zenodo*; 10.5281/zenodo.4282615
Data file 2	donor_health_data	.xlsx	*Zenodo*; 10.5281/zenodo.4282615
Data file 3	otu_table	.tsv	*Zenodo*; 10.5281/zenodo.4282615
Data file 4	metadata	.csv	*Zenodo*; 10.5281/zenodo.4282615
Data file 5	jsd	.pdf	*Zenodo*; 10.5281/zenodo.4282615
Data file 6	pcoa	.pdf	*Zenodo*; 10.5281/zenodo.4282615

The dataset described below is sourced from stool donors from a large, non-profit stool bank (OpenBiome, Cambridge, MA). The bank uses a rigorous screening process [1] that includes (i) an online pre-screen survey where candidates are excluded based on common criteria including body mass index, logistic constraints, and recent antimicrobial use; (ii) an in-person clinical assessment and interview where candidates are excluded for reasons like medication use, infectious disease risk factors, and potentially microbiome-mediated indications such as psychiatric illness; and (iii) a battery of laboratory tests to confirm health status. This results in an average of 3% of candidates accepted as donors [3].

This dataset will complement and extend previously-published sequencing from a subset of the bank’s donors [4, 5]. This dataset will be important for understanding how microbiome communities vary across different populations and contribute to health and disease. We are making it available for use by the scientific community for use on its own or as a healthy control comparison population in studies of disease.

## Data description

As a result of the extensive screening, this population is healthier compared to other sequenced healthy populations like the Human Microbiome Project or the American Gut Project [6, 7]. The criteria used by these large projects describe different portions of the healthy population but do not screen out as many participants as universal stool banks. A full comparison of these criteria is included in Data File 1. The 86 stool donors that have provided these samples are 71% male and 29% female. Their average age is 27.7, and their average body mass index is 23.1. A full table of available donor health data is in Data File 2.

This dataset consists of 200 samples that have been characterized using 16S rRNA sequencing. These samples come from 170 unique donations from 86 individual donors and were sequenced on 11 sequencing runs. Donations from 48 donors were sequenced more than once. Some of these samples have been included as replicates on the same or on different sequencing runs. 11 donations from 9 donors were sequenced more than once on the same run, and 15 donations from 10 donors were sequenced more than once on different runs.

The samples were sequenced by the University of Michigan DNA Sequencing Core on an Illumina MiSeq. The resulting fastq files (Data set 1) were processed using Qiime 2 (version 2020.8) [8] to create an OTU (operational taxonomic unit) table (Data File 3). Briefly, forward and reverse reads were demultiplexed, joined (using *vsearch join*-*pairs* with default settings), quality filtered (using *quality*-*filter q*-*score* with default parameters), and denoised using Deblur (using *deblur denoise*-*16S* with a trim length of 253 bp and minimum requirement of 1 read per sequence) [9]. Taxonomies were assigned to unique sequences using a naïve Bayesian classifier [10] trained on the 99% OTUs in the Greengenes database (version 13_8, using *feature*-*classifier classify*-*sklearn*) [11–13]. Beta diversity was computed using the Jensen-Shannon divergence (using *diversity beta* with 1 pseudocount). Data File 4 is a metadata file describing these samples. 3 samples did not have any denoised reads and were discarded from downstream analysis.

To confirm that the community composition of each donor remains consistent between sequencing runs, we examined the beta diversity between samples from the same donor but different runs, from the same run but different donors, and from the same donor and run. Samples from the same donor but different runs were more similar to one another relative to samples from different donors sequenced on the same run (medians of 0.608 vs. 0.612, *p *= 0.03, Mann–Whitney *U* test; Data file 5). Furthermore, donors explained more of the observed beta diversity than sequencing runs (*R*^2^ 0.72 vs. 0.02, PERMANOVA by marginal effects; Data file 6), confirming that donor microbiota composition remains stable over time and the biological and technical replicates in this dataset.

## Limitations

Although this is a unique and high-quality dataset, no comparator samples from other populations were sequenced along with these samples, so we cannot compare the bank’s stool donor population with the healthy community in general or with any specific disease state. Furthermore, only a subset of samples was sequenced multiple times; a more robust dataset would have additional biological and technical replicates.

## Data Availability

The data described in this Data Note can be freely and openly accessed on the *European Nucleotide Archive* under accession https://identifiers.org/ena.embl:PRJEB41316 [14] and on the *Zenodo* repository under 10.5281/zenodo.4282615 [15]. See Table 1 for details and links to the data.

## Abbreviations

OTU: Operational taxonomic unit
